# Complete mitochondrial genome of *Ischnura asiatica* (Brauer, 1865) assembled from next-generation sequencing data

**DOI:** 10.1080/23802359.2023.2181651

**Published:** 2023-02-28

**Authors:** Keon-Young Jeong, Jong-Yun Choi, Hyunbin Jo, Kwang-Seuk Jeong

**Affiliations:** a Department of Companion Animal Health, Dongju College, Busan, Republic of Korea; b Division of Ecological Survey, National Institute of Ecology, Republic of Korea; c Institute for Environment and Energy, Pusan National University, Busan, Republic of Korea; d Department of Nursing Science, Dongju College, Busan, Republic of Korea

**Keywords:** Complete mitochondrial genome, *Ischnura asiatica;*, Coenagrionidae

## Abstract

*Ischnura asiatica* (Brauer, 1865) is a freshwater damselfly belonging to the family Coenagrionidae that is distributed across most of Korea, primarily in areas with low water flow, such as ponds and wetlands. The complete mitochondrial genome of *I. asiatica* was sequenced by next-generation sequencing. The circular mitochondrial genome was found to be 15,769 bp long, with of 13 protein-coding, two ribosomal RNA, and 22 transfer RNA genes (GenBank accession no. OM310774). Maximum likelihood, phylogenetic analysis showed that this species clustered with other species belonging to the family Coenagrionidae. This study contributes to the phylogeny of damselflies and other members of the family Coenagrionidae.

*Ischnura asiatica* (Brauer, 1865) is a freshwater invertebrate belonging to the family Coenagrionidae. It is distributed across most of Korea, primarily in areas with low water flow, such as ponds and wetlands (Lee et al. 2018), and is an important primary consumer in shallow lake ecosystems (Van de Meutter et al. 2005; Culler et al. 2014). This invertebrate has a relatively small body size and is prey for fishes and other invertebrates as larvae, while for birds and spiders as adults (Outomuro and Johansson 2015; Srivastava et al. 2020). This species has several morphological adaptations to local environmental characteristics and as a part of its defenses against predators. Molecular sequencing of the mitochondrial genome would contribute significantly to understanding genetic variations in this species. The phylogeny of Coenagrionidae is not well known, except for the cytochrome c oxidase subunit I gene. The complete mitochondrial genome sequence of *I. asiatica* has not yet been elucidated. In this study, we analyzed the mitochondrial genome of *I. asiatica* using next-generation sequencing. These results will be useful in further phylogenetic analyses of the family Coenagrionidae.

*Ischnura asiatica* specimens were collected from a tributary (Bokhacheon) of the Han River, South Korea (37°17′41.80″ N, 127°29′23.20″ E). *Ischnura asiatica* larvae were collected during a 30–40 min period by sweeping over the sediment surface and leaves and stems of aquatic macrophytes using a stainless-steel sampler (30 cm width, 600-µm mesh). The samples were sorted to remove plant leaves, stems, and other debris. Collected specimens were identified to species level according to Yoon (1995) and Kawai and Tanida (2005) and stored in the specimen museum of Dongju College (accession number: DCN-0109002, Kwang-Seuk Jeong, kjeong@gsdongju.ac.kr).

*Ischnura asiatica* specimens were homogenized (using a Precellys® 24D homogenizer; Bertin, Montigny-le-Bretonneux, France), and DNA samples were prepared using a DNeasy Blood & Tissue Kit (Qiagen, Hilden, Germany). Library preparation (Illumina TruSeq DNA PCR-free; Cat. No. 20015963) and assembly (SPAdes, V 3.12), and data preparation for DNA sequencing (101 bp from paired-ends on an Illumina NovaSeq 6000) was performed by Macrogen Inc. (Seoul, Korea). The genome was assembled using MEGA-X software (Kumar et al. 2018). We used maximum likelihood implemented in IQ-TREE software (ver. 1.6.12; Nguyen et al. 2015) to construct the phylogenetic tree using the protein-coding gene sequences. The annotated mitochondrial genome sequence of *I. asiatica* is available in the National Center for Biotechnology Information database (Data Availability Statement).

The circular mitochondrial DNA (mtDNA) of *I. asiatica* is 15,769 bp and consists of 13 protein-coding genes (*atp6, atp8, nad1, nad2, nad3, nad4, nad4L, nad5, nad6, cox1, cox2, cox3,* and *cob*), two ribosomal RNA genes (*rrnL, rrnS*) and 22 transfer RNA genes (*trnA, trnC, trnD, trnE, trnF, trnG, trnH, trnI, trnK, trnL1, trnL2, trnM, trnN, trnP, trnQ, trnR, trnS1, trnS2, trnT, trnV, trnW*, and *trnY*), and the control region (between bps 14,789–15,769). The A + T base composition of the genome is 72.4%, and the A + T content of the genes ranges from 59.1% to 80.6%. The G + T base composition of the genome is 27.6%, and the G + C content of the genes ranges from 19.4% to 40.9%. The most common start codon was ATG, followed by ATT. The most common stop codon was TAA. Using IQ-TREE software, a phylogenetic analysis based on the mitochondrial genome of *I. asiatica* and similar mtDNA sequences from the order Odonata (downloaded from GenBank following a BLASTN search) showed that *I. asiatica* clustered with the clade of other species in the *Ischnura* genus with a bootstrap support value of 94%. It also revealed that the family Coenagrionidae was not monophyletic (Figure 1). Considering that the nuclear genomes in the family Coenagrionidae, to which *I. asiatica* belongs have not been sequenced, the availability of a complete mitochondrial genome sequence for *I. asiatica* will be valuable for future phylogeographical studies of the family Coenagrionidae.

**Figure 1.**
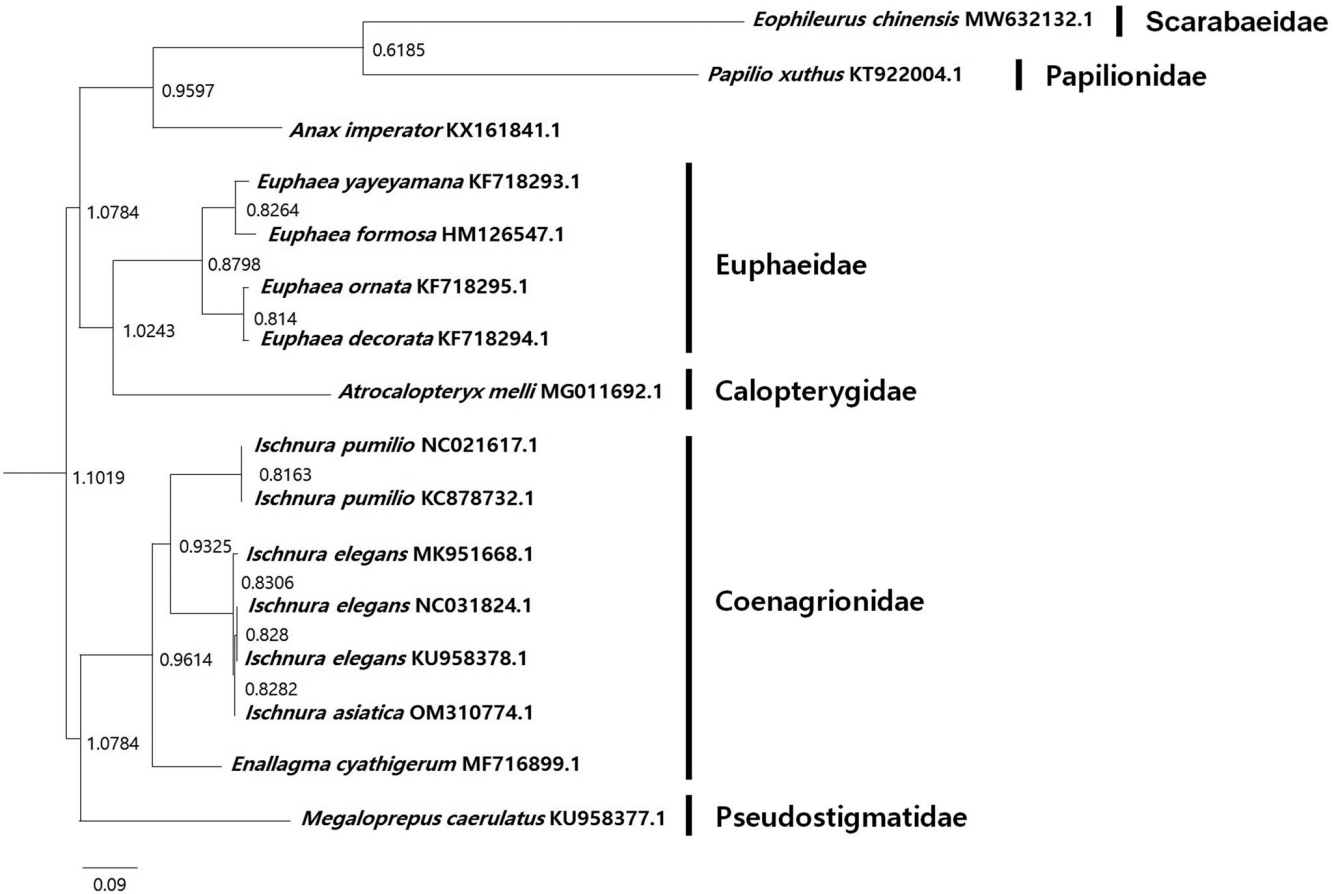
Maximum likelihood phylogenetic tree based on mitochondrial genome sequences.

Recently, climate change is a major driving force in determining species distribution, and Zygoptera (damselflies) are also known to be affected (Ott 2010; Jung et al. 2016). Understanding mitochondrial genome evolution may help researchers prepare strategies for preserving or managing distribution of *I. asiatica* against climate change, including through accurate species identification through molecular barcoding (Rubinoff 2006), or investigation of their energy metabolism (Bratic and Trifunovic 2010).

## Data Availability

The genome sequence data that support the findings of this study are openly available in GenBank of NCBI at https://www.ncbi.nlm.nih.gov/ under accession no. OM310774. The associated BioProject, SRA, and Bio-Sample numbers are PRJNA778563, SRR19369490, and SAMN22964840, respectively.
